# Production of the Plant Hormone Auxin by *Salmonella* and Its Role in the Interactions with Plants and Animals

**DOI:** 10.3389/fmicb.2017.02668

**Published:** 2018-01-12

**Authors:** Clayton E. Cox, Maria T. Brandl, Marcos H. de Moraes, Sarath Gunasekera, Max Teplitski

**Affiliations:** ^1^Department of Soil and Water Science, University of Florida, Gainesville, FL, United States; ^2^Produce Safety and Microbiology Research Unit, United States Department of Agriculture, Agricultural Research Service, Albany, CA, United States; ^3^Smithsonian Marine Station, Ft. Pierce, FL, United States

**Keywords:** indole acetic acid, auxin, RIVET, sprouts, produce safety, *Medicago truncatula*, enteric-plant interactions, tryptophan

## Abstract

The ability of human enteric pathogens to colonize plants and use them as alternate hosts is now well established. *Salmonella*, similarly to phytobacteria, appears to be capable of producing the plant hormone auxin via an indole-3-pyruvate decarboxylase (IpdC), a key enzyme of the IPyA pathway. A deletion of the *Salmonella ipdC* significantly reduced auxin synthesis in laboratory culture. The *Salmonella ipdC* gene was expressed on root surfaces of *Medicago truncatula*. *M. truncatula* auxin-responsive *GH3::GUS* reporter was activated by the wild type *Salmonella*, and not but the *ipdC* mutant, implying that the bacterially produced IAA (Indole Acetic Acid) was detected by the seedlings. Seedling infections with the wild type *Salmonella* caused an increase in secondary root formation, which was not observed in the *ipdC* mutant. The wild type *Salmonella* cells were detected as aggregates at the sites of lateral root emergence, whereas the *ipdC* mutant cells were evenly distributed in the rhizosphere. However, both strains appeared to colonize seedlings well in growth pouch experiments. The *ipdC* mutant was also less virulent in a murine model of infection. When mice were infected by oral gavage, the *ipdC* mutant was as proficient as the wild type strain in colonization of the intestine, but it was defective in the ability to cross the intestinal barrier. Fewer cells of the *ipdC* mutant, compared with the wild type strain, were detected in Peyer's patches, spleen and in the liver. Orthologs of *ipdC* are found in all *Salmonella* genomes and are distributed among many animal pathogens and plant-associated bacteria of the *Enterobacteriaceae*, suggesting a broad ecological role of the IpdC-catalyzed pathway.

## Introduction

Recurrent outbreaks of gastroenteritis caused by non-typhoidal *Salmonella* and shigatoxigenic *E. coli* and linked to the consumption of sprouts, vegetables, fruits and nuts led to the hypothesis that these enteric pathogens use plants as alternate hosts (Brandl, 2006; Brandl et al., 2013; Hernández-Reyes and Schikora, 2013; Walsh et al., 2014). Both *Salmonella* and *E. coli* can persist in manure, soil and water for at least several weeks or months (Brandl, 2006; Brandl et al., 2013; Martínez-Vaz et al., 2014; Wiedemann et al., 2014; Ongeng et al., 2015), from which, they can get established on plants. *Salmonella* and *E. coli* colonized seedlings from contaminated manure, and following the feeding of the infected plants to snails and mice, these bacteria were detected within animals and shed in their feces (Franz et al., 2008; Semenov et al., 2010). While these studies establish that enteric pathogens can use plants as alternate hosts or vectors, less is known about the mechanisms by which they interact with plants, despite the significant progress in the characterization of interactions between enteric pathogens and plants (Klerks et al., 2007a,b; Hernández-Reyes and Schikora, 2013; Wiedemann et al., 2014; Han and Micallef, 2016; de Moraes et al., 2017). Plants are colonized with diverse assemblages of epiphytes and endophytes, which interact with each other and their plant hosts in a variety of ways. Epiphytes often congregate at preferred sites, such as hydathodes, stomata, lesions, or breaks in the epidermis caused by lateral root emergence, which offer more nutrients and potential routes for internalization (Leveau and Lindow, 2001; Lindow and Brandl, 2003). *Salmonella* has been shown to seek similar niches during plant colonization, as it can establish colonies on plant surfaces and may enter internal tissues under conducive conditions (Brandl and Mandrell, 2002; Dong et al., 2003; Klerks et al., 2007a; Golberg et al., 2011; Gu et al., 2013a). However, *Salmonella* likely faces considerable competition for the colonization of these preferred sites and may benefit from mechanisms that allow it to manipulate plant hosts to increase the availability of growth-conducive sites.

Production of plant hormones, and especially indole-3-acetic acid (IAA), is one of the mechanisms by which plant-associated bacteria and fungi manipulate plant growth and development. Microbial production of IAA alters root architecture to increase availability of nutrients to microbes capable of producing this plant hormone. Microbially derived auxins also contribute to the formation of new organs (such as nodules and galls), suppress plant defenses and regulate virulence in phytobacteria (Lindow and Brandl, 2003). The recognition of the roles auxin signaling in plant-microbial interactions led to the hypothesis that it functions as a reciprocal signal in these relationships (Duca et al., 2014).

The synthesis of auxin is widespread among phytobacteria (Duca et al., 2014; Ludwig-Müller, 2015). At least five bacterial IAA biosynthesic pathways exist. Of these IAA biosynthetic pathways, those proceeding via indole-3-acetamide (IAM) and indole-3-pyruvate (IPyA) are the two most widespread and best characterized. Tryptophan is the general precursor of IAA biosynthesis (Patten et al., 2013). The IAM pathway is a two-step process, conserved in three Kingdoms of Life. In the IAM pathway, tryptophan is first converted by tryptophan-2-monooxygenase (encoded by *iaaM*) into IAM, and then to IAA by IAM hydrolase (encoded by *iaaH*) (Lehmann et al., 2010). The IPyA pathway in bacteria is a three-step process mediated by the conversion of indole-3-pyruvate into indole-3-acetaldehyde by indole-3-pyruvate decarboxylase (encoded by *ipdC*) (Brandl and Lindow, 1996; Brandl et al., 1996). Indole-3-acetaldehyde is then converted into IAA. Production of IAA via IpyA in the common bacterial plant colonist *Pantoea agglomerans* contributes to its epiphytic fitness (Brandl and Lindow, 1996, 1998). A given phytobacterial species may commonly possess more than one pathway, suggesting a high degree of redundancy, which is likely indicative of an important phenotype (Patten et al., 2013). For example, the plant pathogen *E. herbicola* pv. *gypsophilae* harbors both pathways, which appear to function in controlling divergent behaviors. The deletion of *ipdC* reduced the epiphytic fitness of this pathogen during active growth on bean leaves, whereas a mutation in *iaaH* was linked to decreased virulence in *Gypsophila paniculata* (Manulis et al., 1998).

Genomic analysis demonstrates that all salmonellae have a copy of the *ipdC* gene, similar to that found in other γ-proteobacteria. Although the presence of the *ipdC* gene in *Salmonella* genomes has been previously detected (Spaepen et al., 2007), its function in *Salmonella* remains unclear. The production of IAA by *Salmonella*, therefore, could enable epiphytic survival in a manner similar to other phytobacteria and could represent a conserved strategy for colonization of plants as a typical part of its life cycle. In this study, we tested the hypothesis that *S. enterica* produces IAA through the IPyA pathway and that the *ipdC*-mediated IAA production impacts the outcomes of the interactions of *Salmonella* with a model plant, *Medicago truncatula*.

## Materials and methods

### Culture conditions and strain construction

All strains used in this study are listed in Table 1, and primers used for the strain construction are in Table 2. Unless otherwise specified, strains were routinely grown in LB medium (Miller, 1972). Minimal A medium was prepared as in Ludwig-Müller (2015). A kanamycin marked *ipdC* mutant was constructed using Datsenko and Wanner mutagenesis (Datsenko and Wanner, 2000) with primers CEC207 and CEC209. The deletion was confirmed with primers BA505 and CEC135. An unmarked mutant was constructed by removing the FRT-*kanR*-FRT cassette with plasmid pCP20 as in Datsenko and Wanner (2000). The deletion was confirmed with primers CEC134 and CEC135. RIVET reporters were constructed as in Merighi et al. (2005) by placing *tnpR* immediately downstream of the *ipdC* stop codon or by placing *tnpR* immediately downstream of the promoter region in a stream where *ipdC* has been removed between the start and stop codons. Constructs were confirmed with primers CEC134 and BA184 or CEC210 and BA184. Activation of the RIVET reporter is scored as the loss of the tetracycline resistance marker in the recovered cells (this occurs because activation of the promoter of interest leads to the expression of the TnpR recombinase and the resulting excision of the tetracycline-resistance marker encoded within “res” sites that are substrates for TnpR; Merighi et al., 2005). A plasmid carrying constitutively expressed *gfp* reporter driven by the *Salmonella dppA* promoter (Noel et al., 2010) was introduced into strains as needed via electroporation. To ascertain that the genetic manipulations did not result in a growth deficiency, strains were grown in LB broth shake cultures at 22°C and 37°C for 10–24 h and OD600 measurements were taken periodically, at 30 min or 2 h intervals.

**Table 1:** Strains used in this study.

**Strain**	**Relevant genotype**	**Source**
14028	Wild-type *S. enterica* serovar Typhimurium	American type culture collection
JS246	14028 *yje*P8103::*res*1*-tetAR-res*1	Merighi et al., 2005
CEC1002	14028 Δ*ipdC*::FRT-*kanR*-FRT	This Study
CEC2002	14028 Δ*ipdC*	This Study
CEC5002	JS246 P*_*ipdC*_*-*tnpR-lacZY* Δ*ipdC*	This Study
CEC8002	JS246 *ipdC-tnpR-lacZY*	This Study
Plasmids	Relevant feature(s)	Source
pGFP-ON	pGFP *dppA*-GFP	Noel et al., 2010
pKD4	*oriR6K bla rgnB* FRT-*kanR*-FRT (kanR)	Datsenko and Wanner, 2000
pKD46	*repA101ts oriR101 araC P_*araB*_- λRed(γ-β-exo)-tL3*) (ampR)	Datsenko and Wanner, 2000
pCP20	*repA101ts λ_*pR*_*-Flp *ci857* (ampR, kanR)	Cherepanov and Wackernagel, 1995
pCE70	*oriR6K* FRT-promoterless *tnpR*-*lacZYα* (kanR)	Merighi et al., 2005
pCE71	*oriR6K* FRT-promoterless *tnpR*-*lacZYα* (kanR)	Merighi et al., 2005

**Table 2:** Primers used in this study.

**Primer**	**Sequence**	**Use**
BA184	CAAAAAGTCGCATAAAAATTTATCC	RIVET confirmation
K2	CGGTGCCCTGAATGAACTGC	FRT-*kanR*-FRT confirmation Datsenko and Wanner, 2000
CEC134	TCCCCCTGTGGCGTGAAT	*ipdC* confirmation
CEC135	CCTGGCTATTGCTGGCGG	*ipdC* confirmation
CEC207	GCATTCCTTAATACTCAACATAATATCAACGTCAGAAGGAAAGCTGTCtgaggctggagctgctt	*ΔipdC* construction
CEC208	TTACTGCGTACCGTGACCCGGGCGCTGGAAGCCCGCAACGGGGGATAAtgtaggctggagctgctt	*ipdC-tnpR-lacZY* construction
CEC209	TGGCCCCCGCTGCGCCGGATTAGGGTTCGTGACGGTTGGCGGCCAGCAcatatgaatatcctccttag	ipdC_LR
CEC210	GGACAGCCAGTGCGGATT	*ipdC* confrimation

*Merighi et al. (2005), Noel et al. (2010), Datsenko and Wanner (2000), Cherepanov and Wackernagel (1995)*.

### Detection of IAA using salkowski reagent

*Salmonella enterica* sv Typhimurium (*S*. Typhimurium thereafter) cultures were grown overnight from glycerol stocks at 37°C. 1 mL was washed 3x in PBS and was diluted 1,000x in 5 mL Minimal A medium with or without 1 mM tryptophan. Cultures were incubated at 22°C for 72 h. Supernatant was harvested from 1 mL of culture by centrifugation for 15 min at 7,000 × g. The supernatant was transferred to a 12 mm × 75 mm glass tube and 2 mL of fresh Salkowski reagent R1 (Glickmann and Dessaux, 1995) was added and vortexed to mix. The tubes were incubated for 30 min at 30°C in the dark, aliquots were then withdrawn and A540 was measured using the Shimadzu biospec-mini.

### IAA extraction, purification, and identification

Hundred millliter of *Salmonella* culture filtrates were extracted twice with 150 and 100 mL of ethyl acetate. The extracts were rotary evaporated over a water bath at 40°C. The resulting yellow oily residue was brought up in 600 μl of methanol. The extracts and standards (IAA, tryptophol and IPyA) were subject to reverse phase (C_18_) liquid chromatography using Waters Atlantis dC18-5 μM column using isocratic elution (72 water 7.2% acetic acid 20.8% methanol), as before (Brandl and Lindow, 1996; Brandl et al., 1996). Eluting substances were detected with a UV/VIS detector set to 230 and 280 nm (Brandl and Lindow, 1996; Brandl et al., 1996). Low Resolution Electrospray Ionization LCMS was carried in a Thermo Scientific LTQ LC-MS instrument using a Grace Vydac 218TP C18 (5 μ, 7.5 cm, 2.1 mm ID) column. LCMS was run using a gradient of 10% acetonitrile (with 0.1% formic acid) and 90% water (with 0.1% formic acid) to 100% acetonitrile (with 0.1% formic acid) from 0 to 15 min and then 100% acetonitrile with formic acid was continued till 21 min.

### Plant infections

Seeds of *M. truncatula* A17 (wild type) and transgenic line containing the *GH3::GUS* reporter (van Noorden et al., 2007) were surface sterilized in ethanol and then in a diluted chlorox bleach solution as before (Mathesius et al., 2003) and stratified overnight at 4°C. Imbibed seeds were transferred into individual growth pouches and were left to germinate at room temperature overnight. Once radicles reached ~1 cm, six seedlings (per treatment) were inoculated with 100 μl of the suspension containing ~5,000–10,000 CFU of either CEC1000 (*S*. Typhimurium 14028 marked with a kanamycin cassette in a neutral site) or CEC1002. Plants were watered as needed with the N-free Hoagland solution. To detect activation of the *GH3::GUS* reporter, seedlings were withdrawn after 48 hrs and 6 days past infection, and stained with X-gluc (10 mg/ml) in 0.2 mM Phosphate buffer (pH 7.2) overnight. They were then washed 3 times with cold phosphate buffer to remove excess substrate and the seedlings were kept in 50% ethanol/phosphate buffer at 4C. They were imaged under a dissecting microscope Olympus MVX10 fit with a MicroFIRE camera (Optronics).

For the reporter assays, washed RIVET reporter suspensions (containing ~10,000–100,000 CFU) were inoculated onto *M. truncatula* seedlings as described in Supplemental Information. At indicated time intervals, seedlings were excised from pouches, blended with a glass mortar and pestle and dilution plated onto XLD with kanamycin. Bacteria were patched from these onto LB with tetracycline.

To detect changes in lateral root formation, seeds of *M. truncatula* A17 were surface sterilized, imbibed and then transferred into growth pouches. Seedlings were inoculated with 100 μl of the washed bacterial suspension containing ~5,000–10,000 CFU of either CEC1000 (*S*. Typhimurium 14028 marked with a kanamycin cassette in a neutral site) or CEC1002 (*ipdC::kan*).

### Mouse infections

BALB/c mice were inoculated by oral gavage, which results in a systemic infection. Prior to the infections, *S. enterica* sv. Typhimurium 14028 and CEC1002 were streaked on LB agar and incubated at 37°C overnight, colonies were harvested and re-suspended in 1 ml of sterile PBS. Each suspension was adjusted to a final dilution of ~10^8^ CFUs/ml. Groups (*n* = 3) of female BALB/c mice received three doses of *Salmonella* in 0.2 ml of PBS. Inoculum doses were confirmed by serial titration, and were found to be 10^6^, 10^4^, and 10^2^ CFU per injection dose for the wild type, and 2.2 × 10^6^, 2.2 × 10^4^, and 2.2 × 10^2^ CFU per injection for the *ipdC* mutant. Animals were observed daily for 7 days after infection and distressed animals presenting signs of morbidity were euthanized, their organs were not sampled. The experiment was terminated at 7 days, and all surviving animals were sacrificed. Liver, spleen, large intestine, and Peyer's patches were harvested. Tissues were weighed, and homogenized in sterile PBS in TissueLyser II (Qiagen). Bacterial homogenates were serially diluted in PBS and plated onto Xylose-Lysine Deoxycholate (XLD) agar (Beckton, Dickinson and Company) plates, followed by incubation at 37°C overnight for bacterial CFU counts. All animal care and procedures were in accordance with institutional policies for animal health and well-being and approved by the University of Florida Institutional Animal Care and Use Committee (IACUC).

### Data analysis

Synteny analysis was carried out using default parameters in the SyntTax web server (Oberto, 2013). Sequences were imported from GenBank. UV peak integration was conducted with proprietary Waters software, and analysis of mass spectral data was carried out using Thermo Scientific proprietary software. Means and standard error of the mean were calculated in Microsoft Excel v. 14.0.0.

## Results

**Figure 1:**
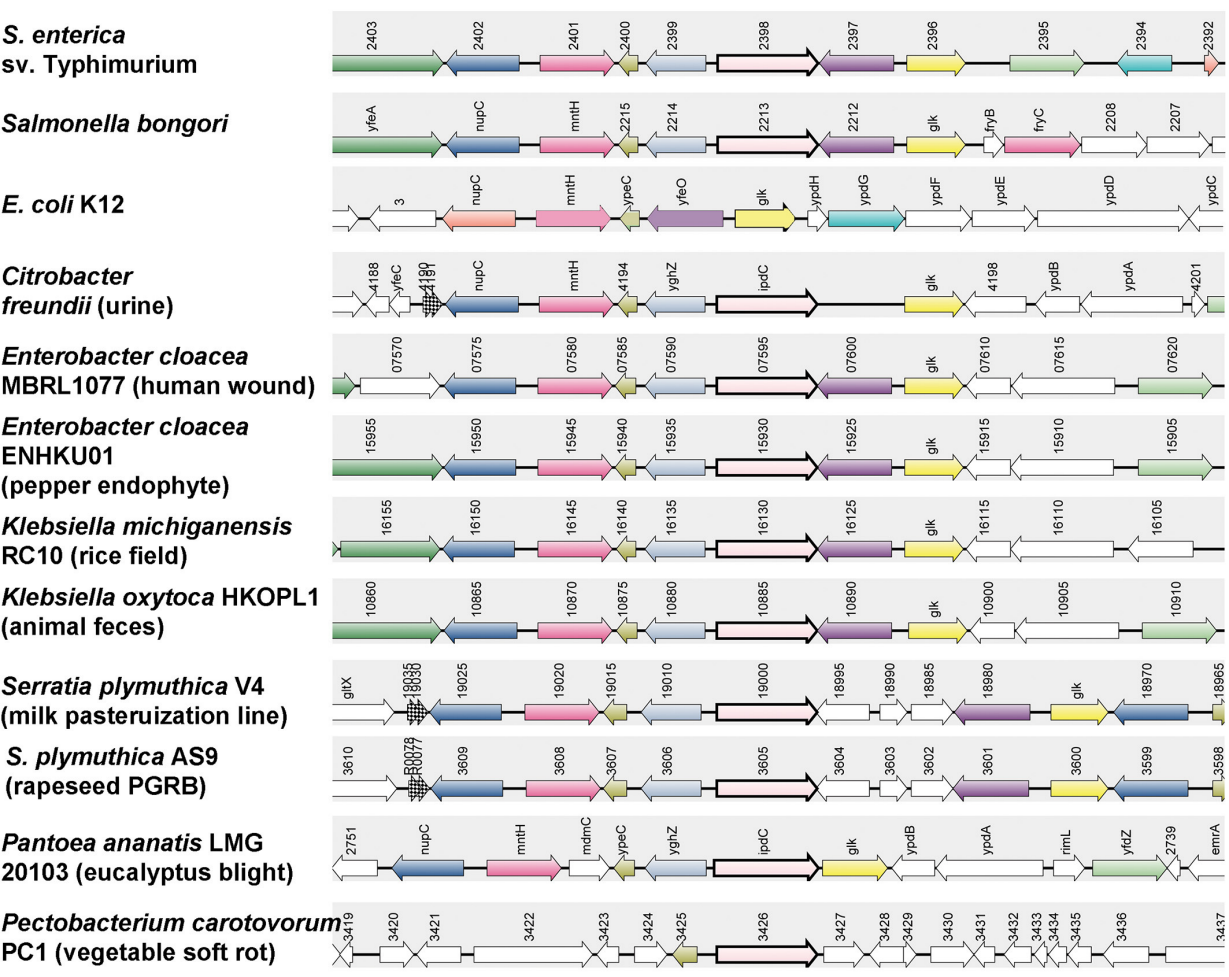
Synteny analysis of *ipdC* in *Enterobacteriaceae*. Genes homologous to the *Salmonella ipdC* were retrieved from NCBI. Synteny analysis was carried out using SyntTax (Oberto, 2013). Orthologs of *ipdC* are shown as pink arrows with a thick black border.

*ipdC is conserved in salmonellae. ipdC*, the gene encoding indole-3-pyruvate decarboxylase, a key enzyme in the IPyA pathway, is common in many members of *Enterobacteriaceae*; including organisms that are commonly associated with animals as well as with plants. Genomic analysis showed that all available *Salmonella* genomes harbor *ipdC*, but that it is absent in *E. coli, Shigella*, or *Erwinia* genomes and in some strains of *Enterobacter*. The presence or absence from the enterobacterial genomes does not seem to correlate with the source of the isolates, and *ipdC* was as likely to be found in the genomes of animal as of plant isolates of this genus. Synteny analysis revealed a distribution of orthologs in the genomes of many strains belonging to the *Enterobacteriaceae* where it is generally found within similar genomic context (Figure 1). In *Salmonella, ipdC* is located downstream of an ortholog of the glucokinase *glk*, and a putative ion channel protein known as YfeO. *glk* was conserved at this location in all genomes except in *Pectobacterium*, in which it is present at another site. Similarly, YfeO is present at different sites in *Citrobacter, Panteoa* and *Pectobacterium* genomes. Immediately downstream of *ipdC* in the *Salmonella* genome are an ortholog of the L-glyceraldehyde 3-phosphate reductase *gpr*, and an uncharacterized periplasmic protein YpeC. Although its function is currently unknown, *ypeC* was present at a similar location in all genomes. *gpr* (*yghZ*) was present between *ipdC* and *ypeC* except in *E. coli* and *Pectobacterium*. These results suggest that the *Salmonella ipdC* may encode an ortholog of a well-characterized indolepyruvate decarboxylase, responsible for IAA synthesis in other bacteria. Therefore, we tested whether *ipdC* is capable of directing IAA production in *S. enterica* sv Typhimurium.

### IAA production by *Salmonella* via *ipdC*

**Figure 2:**
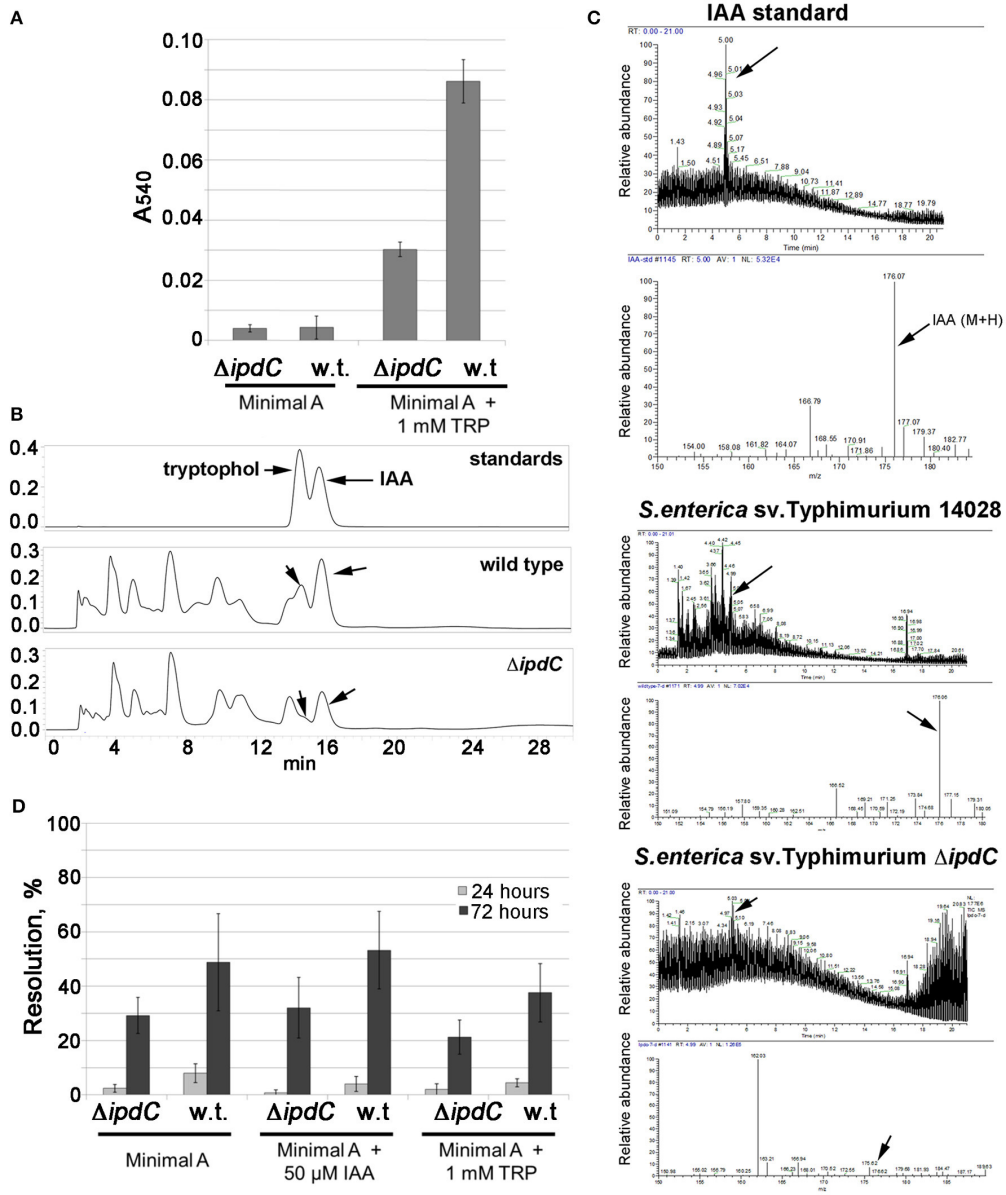
Production of IAA in *ipdC*-dependent manner by *Salmonella enterica* sv. Typhimuirum 14028. **(A)** Detection of IAA by the Salkowski reagent. Results are from replicates from three independent experiments, averages are shown. Error bars are standard deviations. **(B)** HPLC detection of IAA and tryptophol in spent cultures of the wild type and the *ipdC* mutant. Hydrophobic fractions of *Salmonella* culture filtrates were subjected to reverse phase (C_18_) liquid chromatography, eluted isocratically with acidified water/methanol and eluting substances were detected with a UV/VIS detector set to 230 and 280 nm. **(C)** Low Resolution Electrospray Ionization LC-MS of the hydrophobic fraction of *Salmonella* culture filtrates. LTQ LC-MS was carried out using a Grace Vydac 218TP C18 and acetonitrile/water gradient. **(D)** Expression of the *ipdC* RIVET reporter in the wild type and Δ*ipdC* backgrounds was measured in Minimal A medium with and without synthetic IAA or tryptophan in cultures incubated at 22°C for 72 h. Samples were streaked to xylose lysine deoxycholate (XLD) agar with kanamycin at 24 and 72 h. Plates were incubated at 37°C overnight and colonies were patched to LB agar with tetracycline to quantify resolution. Experiments were repeated five times (without technical replications), averages from the five experiments are shown. Error bars are standard deviations.

The ability of *Salmonella* to produce IAA was first tested in culture in Minimal A medium using a colorimetric assay based on the Salkowski reagent. While there was no auxin production by either the wild type or the isogenic *ipdC* mutant grown in unammended Minimal A medium, production of IAA was detected in the medium supplemented with 1 mM L-tryptophan, the precursor for the IPyA pathway (Figure 2A). The intensity of absorbance at 540 nm after addition of the Salkowski reagent, which correlates with the presence of indole compounds, including IAA, was significantly lower in the culture filtrate extract of the *ipdC* mutant than that of the wild type strain (Figure 2A). When culture filtrate extracts were separated using HPLC, UV peaks corresponding to IAA and tryptophol were observed for the wild type, but were strongly reduced for the *ipdC* mutant (Figure 2B). Tryptophol is a product of a reduction of indole-3-acetaldehyde, the product of the reaction catalyzed by *ipdC*. The production of IAA by the wild type *S. enterica* sv. Typhimurium 14028 was confirmed by LC-MS/MS. Production of IAA was significantly reduced (but not completely eliminated) in the isogenic strain lacking *ipdC* (Figure 2C). In laboratory cultures, expression of the *ipdC* RIVET (recombinase-based *in vivo* expression technology) reporter was low (less than 10%) after 24 h of incubation, however, it increased by 72 h (Figure 2D). Consistent with the production of IAA in laboratory media, a recombinase *in vivo* expression technology (RIVET) reporter in *ipdC* was expressed in a laboratory medium at 45–55% (Figure 2D). Expression of the reporter in the *ipdC* background was considerably lower than in the wild type, suggesting a feedback regulation. However, supplementation of the medium with 50 μM IAA did not significantly affect expression of the *ipdC* RIVET reporter in the wild type or in the *ipdC* mutant. Despite clear differences in the amount of IAA produced in a minimal medium with and without tryptophan, the *ipdC* RIVET reporter was expressed at similar levels in the minimal medium with and without tryptophan (Figure 2D), suggesting that the availability of the substrate is critical for the function of the IpdC enzyme (as indirectly evidenced by the accumulation of IAA in culture) but not for transcription of the encoding gene.

### Plant responses to IAA from *Salmonella*

**Figure 3:**
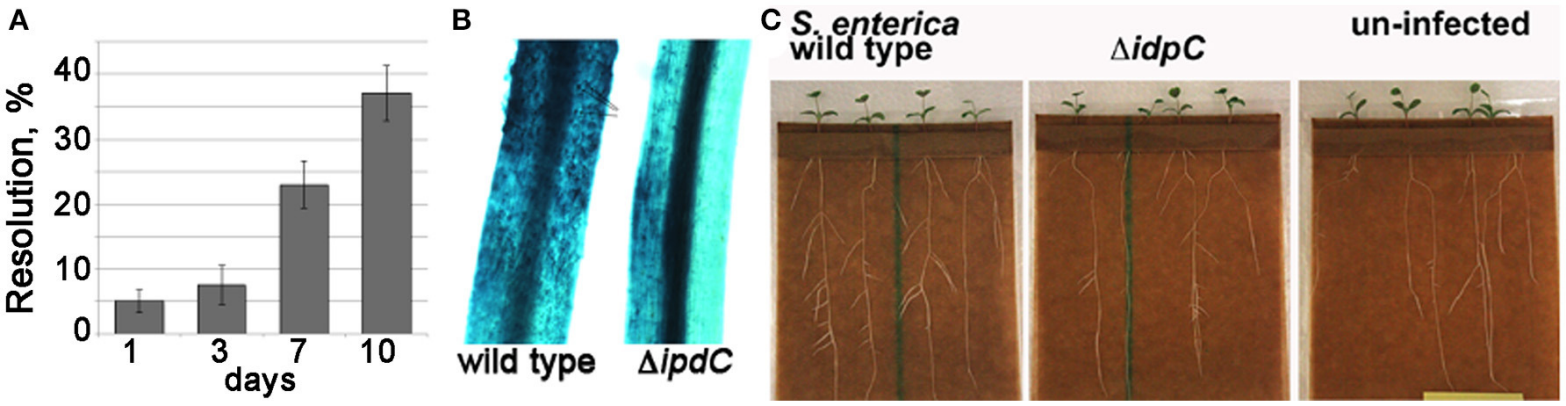
*M. truncatula* responds to the activation of *ipdC* in the rhizosphere. **(A)** Resolution of the *Salmonella ipdC* RIVET reporter was measured on the seedlings grown in pouches from surface-sterilized *M. truncatula* Jemalong A17 seeds. **(B)** Activation of the *M. truncatula* auxin-responsive *GH3::GUS* reporter was measured as in Mathesius et al. (2003). Black arrows point to representative sites of *GH3::GUS* expression in the cortex, likely a result of the IAA production by the wild type *Salmonella*. Six seedlings were inoculated per treatment, and results of a representative experiment (6 days post- infection) are shown. Care was taken to excise root segments at the same developmental stage **(C)** Inoculation with the wild type *Salmonella* Typhimurium 14028 increases lateral root formation in *M. truncatula* Jemalong A17.

To determine whether *Salmonella* produces auxin on plant surfaces and whether this bacterially-produced plant hormone has a function during colonization of plant hosts, *ipdC* expression was assessed in the *M. truncatula* rhizosphere. The *ipdC* RIVET reporter was resolved at ~5–8% during the first 3 days of root colonization, reaching 22 and 37% at seven and 10 days post-inoculation, respectively (Figure 3A). When compared with the expression of the same reporter in Minimal A medium (Figure 2D), it does not appear that *ipdC* is expressed stronger in the rhizosphere, nevertheless, it is clear that *ipdC* is expressed in *S*. Typhimurium on roots during colonization. Next, we tested the consequences of the *ipdC* mutation using a *GH3::GUS* reporter in *M. truncatula*. Genes belonging to the *GH3* (Gretchen Hagen 3) family are among the regulators that control the dynamic process of endogenous auxin homeostasis (Yang et al., 2015). This *GH3::GUS* reporter is normally expressed in the stele of the root, while expression in the cortical cells results from exposure to exogenous IAA, including that produced by bacteria such as by the symbiotic *Sinorhizobium meliloti* (Mathesius et al., 1998, 2000; van Noorden et al., 2007). As shown in Figure 3B, the *GH3*::*GUS* reporter was broadly induced in the cortical tissue of *M. trucatula* roots after inoculation with the *S*. Typhimurium wild type strain, but only at discrete locations in the cortex after that with the *ipdC* mutant. Taken together, the activation of the plant *GH3::GUS* reporter by *Salmonella* and the expression of the *Salmonella ipdC* gene in the rhizosphere suggest that this human pathogen synthesizes auxin via IpdC during plant colonization and in quantities that are detectable by the plant cells and sufficient to cause changes in plant gene expression.

### Production of IAA creates a niche for *Salmonella*

**Figure 4:**
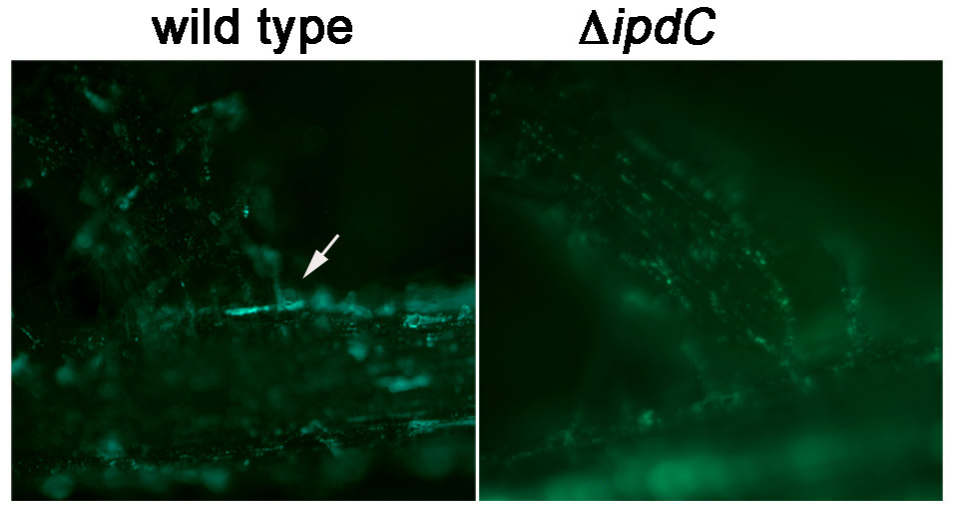
Colonization of sites of lateral root emergence by *Salmonella* wild type and Δ*ipdC*. *M. truncatula* seedlings were inoculated with either CEC1000 or CEC1002 labeled with pGFP-ON. Fluorescence of the bacteria on roots of seedlings was documented 12 days after inoculation using Olympus BX41 epifluorescent microscope with 3.3 MPX Olympus camera. The white arrow points to the microcolony of the wild type *Salmonella* at the base of the lateral root.

To determine how *Salmonella* may benefit from the production of IAA, we first tested whether perception of the bacterially-produced IAA led to observable phenotypic changes in the inoculated plants. Secondary root initiation is a phenotype known to be regulated by IAA (Spaepen et al., 2014; Bensmihen, 2015). Therefore, we evaluated formation of secondary roots in the seedlings inoculated with the wild type *Salmonella* or the isogenic *ipdC* mutant. As shown in Figure 3C, few secondary roots had emerged in 2-week old *M. truncatula* seedlings that were not inoculated with *Salmonella* and treated with the nutrient solution only (control). Seedlings inoculated with the *S*. Typhimurium wild type strain showed 5–12 secondary roots, while seedlings inoculated with the *ipdC* mutant had 2–7 secondary roots per plant. When the wild type and the *ipdC* mutant were tagged with a plasmid constitutively expressing GFP, the *ipdC* mutant was found to be evenly distributed throughout the root surface and on root hairs, while the wild type tended to form large aggregates at the sites of the lateral root emergence (Figure 4). However, we did not observe differences in the total number of bacteria recovered from the seedlings (data not shown). In laboratory LB shake cultures, the wild type and the *ipdC* mutant grew with the same kinetics and to the same final optical densities at 22 and 37°C.

### Deletion of *ipdC* reduces virulence in the mouse model

**Figure 5:**
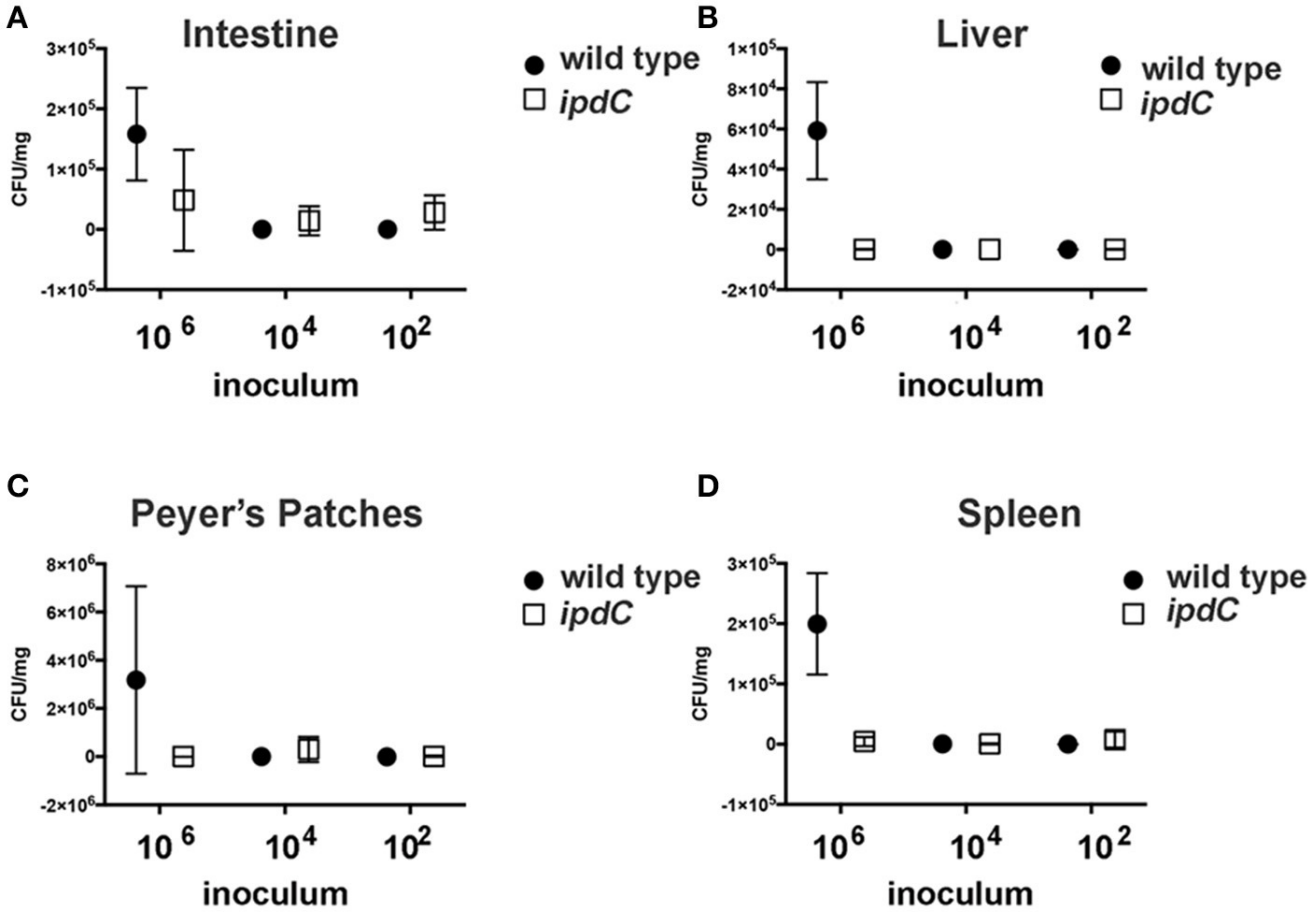
Recovery of *Salmonella* from mouse organs. Three female BALB/c mice per three infectious doses were infected with the wild type *S*. Typhimurium 14028 or an isogenic *ipdC* mutant by oral gavage. After 7 days, surviving animals were euthanized, and organs harvested for the determination of bacterial load. Recovered CFU of the mutant (empty square) and the wild type (filled circle) are shown. Error bars are standard deviations. When error bars are not shown, this indicates that only a single animal was remaining upon completion of the experiment. *Salmonella* recovery from intestine **(A)**, liver **(B)**, Peyer's patches **(C)** and spleen **(D)** is shown.

We hypothesized that because IAA is a plant hormone, the function of *ipdC* may be confined to the interaction of *Salmonella* with its plant hosts. To test this hypothesis, the comparative ability of the *S*. Typhimurium wild type and *ipdC* mutant to colonize mice following an oral gavage was determined. The hypothesis that the mutation in *idpC* impacts virulence in mice was proven null (Figure 5). While the *ipdC* mutant did not differ from the wild type in its ability to establish within the intestine, it was deficient in the colonization of the liver and the spleen (Figure 5). However, due to the small number of animals infected per dose, robust statistical analyses of these data were not possible.

## Discussion

The presence of *ipdC* in *Salmonella* was first noted by Spaepen et al. (2007) while constructing a phylogenic analysis to support the differentiation of the *ipdC* product of *Azospirillum brasilense* as a phenylpyruvate decarboxylase in contrast to the classical indole-3-pyruvate decarboxylase product of *ipdC* in *Enterobacter cloacae* (Spaepen et al., 2007). Of the 33 species with genomes containing putative *ipdC* homologs considered in their analysis, *ipdC* of *S*. Typhimurium LT2 grouped independently but was most closely related to *E. cloacae* and *Pseudomonas putida*, the strains most distant from *A. brasilense*, supporting the hypothetical function of *Salmonella ipdC* in synthesis of auxin. Our analysis of genomes in the currently available databases, revealed that *ipdC* is present in all *Salmonella* genomes, including that of the obligate human pathogen *Salmonella* Typhi. The synteny analysis presented here confirms also the common presence of *ipdC* in the genomes of both human and plant pathogenic *Enterobacteriaceae*.

The results of this work suggest that the *Salmonella ipdC* contributes to the production of IAA in laboratory cultures, that this gene is expressed in laboratory media and during root colonization, and that the product of the reaction involving IpdC activates plant auxin-responsive promoter. Inoculation of *M. truncatula* seedlings with the wild type *Salmonella*, but not that with the *ipdC* mutant resulted in increased production of lateral roots. Although the wild type and the *ipdC* mutant were recovered from the seedlings in similar numbers, the wild type cells tended to cluster at the sites of lateral root emergence, where high tryptophan availability to bacterial colonizers was reported with a whole-cell biosensor (Jaeger et al., 1999), whereas cells lacking *ipdC* were evenly distributed in the rhizosphere. This indicates that *Salmonella* cells located at sites where tryptophan is likely abundant benefit from IAA production to achieve greater cell densities. (Brandl and Lindow, 1998) suggested that IAA production by bacterial plant colonists may increase their fitness by enhancing nutrient leakage from plant cells by cell wall remodeling (Brandl and Lindow, 1998). Collectively, these results point at the ability of *Salmonella* to manipulate their plant hosts through IAA production although the entire suite of plant functions subject to this manipulation is not yet known. Plant pathogens, epiphytes, symbionts and endophytes employ auxin to thwart plant defenses, create new organs (nodules and galls), alter plant tissue development, and induce leakage of nutrients (Lindow et al., 1998; Lindow and Brandl, 2003; Duca et al., 2014; Talboys et al., 2014; Ludwig-Müller, 2015). While we present evidence that *Salmonella* expresses *ipdC* in the plant rhizosphere and that plants sense and respond to the resulting presence of this exogenous auxin, the broader implications of our observations for the ecology of *Salmonella* on plants remain unknown. In interpreting the results of this study, it is important to recognize that they were conducted in growth pouches that are largely devoid of native phytomicrobiota, which can strongly influence the outcomes of interactions between human enteric pathogens and plants (Klerks et al., 2007a; Teplitski et al., 2011; Brandl et al., 2013; Gu et al., 2013b). It is not clear whether production of IAA by *Salmonella* is of consequence during interactions under the field conditions, in natural soils where *Salmonella* must compete with other rhizosphere microbes, many of which produce IAA as well. In natural environments, effects of IAA on plants are concentration-dependent: low concentrations could induce formation of lateral roots, suppress host defense responses, while at higher doses it can inhibit plant growth (Kazan and Manners, 2009; Duca et al., 2014; Ludwig-Müller, 2015). It is clear that the production of IAA is dependent on the availability of tryptophan. In the rhizosphere, bacteria that produce more tryptophan, rather than depend on exogenous sources, can produce more IAA and therefore tend to have an advantage (Duca et al., 2014). At least initially, in a gnotobiotic rhizosphere *Salmonella* appears to utilize tryptophan that is exuded from *M. truncatula* seedlings. It is not certain that *Salmonella* can scavenge sufficient tryptophan or synthesize it *in situ* when it competes with the native rhizosphere microorganisms. Therefore, the ecological role of *Salmonella*-synthesized IAA in natural interactions with plants remains to be elucidated.

The role for *ipdC* during *Salmonella* mouse infection was unexpected. We initially hypothesized that the production of IAA must represent a unique adaptation to the plant-associated lifestyle. Upon further reflection, it should come as no surprise that the same molecule may have beneficial functions in a number of environments, where successful opportunists (such as non-typhoidal *Salmonella*) have evolved to thrive. Studies suggest that in some bacteria, IAA aids in stress adaptation and protection against stressors such as UV, salt and acidity (Duca et al., 2014). It is possible that these functions of IAA explain the mouse phenotype of the mutant. It is also possible that the phenotype of the *ipdC* mutant in the mouse may be—at least partially—independent of IAA and rather dictated by other functions of IpdC. For example, while indolpyruvate decarboxylases are generally known to have high affinity for indolpyruvate, they can also use phenylpyruvate, pyruvate and benzoylformate as substrates. It is, therefore, possible that IAA production is not the main, but rather incidental, role for these enzymes in some organisms (Duca et al., 2014). The conservation of *ipdC* in many bacterial animal pathogens that behave as opportunistic plant colonists and, reversely, in epiphytic bacteria that opportunistically colonize animal tissue, provides new incentives to gain insight into the function of this plant hormone in a larger biological context.

## Ethics statement

Results of mouse experiments are described. Animal protocols were reviewed and approved by the University of Florida IACUC.

## Author contributions

CC, MB, and MT conceived the project and designed the experiments, CC and MT carried out experiments and data analyses in Figures 1, 2A,B,D, 3A, 4. SG carried out chemical identification of IAA (Figures 2B,C). MdM designed and conducted mouse experiments.

### Conflict of interest statement

The reviewer AHCVB declared a shared affiliation, with no collaboration, with several of the authors (CC, MdM, and MT) to the handling Editor. The other authors declare that the research was conducted in the absence of any commercial or financial relationships that could be construed as a potential conflict of interest.
